# Neopterin Levels in Periodontitis and after Nonsurgical Periodontal Therapy: Evaluation of Gingival Crevicular Fluid, Oral Fluid, Serum and Urinary Samples—A Case-Control Study

**DOI:** 10.3390/biomedicines10123200

**Published:** 2022-12-09

**Authors:** Ondrej Heneberk, Andrea Vernerova, Lenka Kujovska Krcmova, Eliska Wurfelova, Vladimira Radochova

**Affiliations:** 1 Department of Dentistry, Faculty of Medicine in Hradec Kralove, Charles University, 500 03 Hradec Kralove, Czech Republic; 2 Department of Dentistry, University Hospital Hradec Kralove, Sokolská 581, 500 05 Hradec Kralove, Czech Republic; 3 Department of Analytical Chemistry, Faculty of Pharmacy, Charles University, 500 05 Hradec Kralove, Czech Republic; 4 Department of Biochemistry and Diagnostics, University Hospital Hradec Kralove, 500 05 Hradec Kralove, Czech Republic

**Keywords:** neopterin, periodontitis, macrophages

## Abstract

Periodontitis is a chronic inflammatory disease that leads to the destruction of the tooth-supporting tissues with complex immune response. Neopterin (Np), secreted via activated macrophages, is considered a biomarker of cellular immunity. The aim of this study was to evaluate the impact of periodontitis and nonsurgical periodontal therapy. Np gingival crevicular fluid (GCF), oral fluid, serum and urine levels were compared in subjects with periodontitis before periodontal treatment, three months after and in a healthy control. Np GCF concentrations in the study group after treatment were significantly higher than the control group (*p* = 0.038). The GCF total amount (amount of substance) was significantly higher in the study group before periodontal treatment than in the control group (*p* = 0.001) and higher than the levels taken after treatment collection (*p* = 0.024). The oral fluid Np concentrations in the study group after treatment were significantly increased compared to the before treatment concentrations (*p* = 0.020). The same trend was observed in the urine samples. Significant correlation was found between the serum and oral fluid Np concentrations (*p* = 0.001, ρ = 0.40). Our results confirm the impact of cellular immunity and macrophages on periodontitis and on the resolution of periodontal inflammation. The presence of neopterin in oral fluid most likely originates in the serum.

## 1. Introduction

Periodontitis is a chronic multifactorial inflammatory disease associated with oral dysbiotic plaque biofilms and is characterized by the progressive destruction of the teeth-supporting apparatus [1]. The development of periodontitis results from a complex interaction between the biofilm and the immune system. It is a host response that is responsible for an 80% risk of tissue destruction [2,3].

Cellular immunity, both innate and adaptive, plays an important role in periodontitis. of the conclusions drawn by previous studies are not consistent in regard to whether cellular immunity contributes more to the progression of periodontal lesions or to the establishment of a stable lesion [4,5,6]. Macrophages are resident cells in the periodontal tissue with a phagocytic ability. They contribute to connective tissue damage and bone resorption through the release of matrix metalloproteinases, collagenases, and proinflammatory cytokines [6,7,8].

Neopterin (Np) is a metabolite of guanosine triphosphate (GTP) [9,10]. It is released by activated monocytes/macrophages after stimulation via interferon gamma (IFN-γ) that is released mainly via activated T helper subtype 1 lymphocytes and natural killer cells. Np levels were found to be correlated with IFN-γ levels. Thus, Np is considered a biomarker of IFN-γ and the activation of overall cellular immunity [8,10].

Np has been studied in periodontitis by several studies in gingival crevicular fluid (GCF) [8,9,11,12,13], oral fluid (OF) [8,13,14,15,16], serum [11,17,18] and urine [8,13,16,17]. Np was proposed for the monitoring of periodontal disease activity [12], but the complex evaluation of Np levels in these biologic fluids, including the impact of nonsurgical periodontal therapy, is yet to be studied.

The purpose of our study is to compare the levels of Np in GCF, oral fluid, serum, and urine in subjects with periodontitis to the healthy control and to evaluate the impact of nonsurgical periodontal therapy on the levels of Np in these biological fluids. The second aim is to compare Np levels among these matrices using correlation analysis to find possible relations between Np levels in these body fluids.

## 2. Materials and Methods

### 2.1. Study Design

50 subjects of both genders were recruited from outpatients of the Department of Dentistry of the University Hospital Hradec Kralove, Czech Republic. Based on our preliminary observations, where the GCF mean ± standard deviation Np was 72.85 ± 25.65 nmol/L, and 30% expected Np levels changes, a test power of 80% and an alpha of 5%, the minimum number of subjects to be recruited was calculated to be 22 for each group. The study group consisted of 25 subjects who fulfilled the criteria of periodontitis, according to the classification of the American Academy of Periodontology and European Federation of Periodontology [1]: (a) Interdental clinical attachment loss (CAL) detectable at ≥2 non-adjacent teeth; or (b) Buccal or oral CAL ≥ 3 mm with pocketing ≥3 mm detectable at ≥2 teeth. However, the observed CAL cannot be ascribed to non-periodontitis-related causes such as: gingival recession of traumatic origin; dental caries extending in the cervical area of the tooth; the presence of CAL on the distal aspect of a second molar and associated with malposition or extraction of a third molar; an endodontic lesion draining through the marginal periodontium; and the occurrence of a vertical root fracture [1]. One individual did not complete the periodontal therapy, so he was excluded from after treatment evaluation. The control group included 25 subjects who presented with clinically and radiologically healthy periodontium. The gender and age of these individuals matched the gender and age ratios of the control group. All subjects included in the study were systematically healthy adults and at least 18 years old. Subjects with any intraoral mucosal lesions, smokers, and edentulous subjects were excluded.

### 2.2. Dental Examination

The periodontal examination was performed at the Department of Dentistry, University Hospital Hradec Kralove, Czech Republic. Two experienced physicians, H.O. and R.V., were trained to provide treatment using the approved protocol for this study. The reliability between examiners was evaluated by using kappa coefficients before the start of the study. Gingival inflammation was evaluated using the gingival index (GI), adapted from Löe and Silness [19]. The plaque index (PLI) was adopted according to the method published by Silness and Löe [20]. Periodontal status was determined with periodontal pocket depth (PPD), CAL, gingival recession (GR). A periodontal probe with fine calibration by single-millimeter grading (PCPUNC156, HU - Friedy, Chicago, IL, USA) was used for that evaluation. Data from each fully erupted tooth were obtained from six aspects (mesiovestibular, vestibular, distovestibular, distooral, oral, mesiooral) and the mean value was calculated for each tooth sample. The third molars and the retained roots were omitted. For the full mouth evaluation, the mean GI, PLI, PPD, GR, and bleeding on probing (BOP) were calculated. The decayed—missing—filled (DMF) tooth index was recorded for each subject [21,22]. A decayed tooth presented with at least one caries, while a filled tooth with at least one filled surface; missing teeth were also reported [22]. Radiographic bone loss was assessed via an orthopantomogram for each individual. The dental examination was performed 72 h prior to the collection of each sample to avoid blood contamination of the sample resulting from bleeding on probing [23].

### 2.3. Sample Collection, Preparation, and Analysis

The samples (GCF, oral fluid, urine, and serum) were collected in the study before periodontal therapy and in control group. In the study group, the second samples were collected 3 months after nonsurgical periodontal therapy, due to the minimum 8 week period of periodontal lesion healing [24]. All samples were collected early in the morning, after fasting overnight. Upon collection, the samples were immediately sealed in a sun-protected box and transported to the research laboratory of the Department of Clinical Biochemistry and Diagnostics of the University Hospital Hradec Kralove (Czech Republic) for samples extraction, storage and analysis.

**Figure 1:**
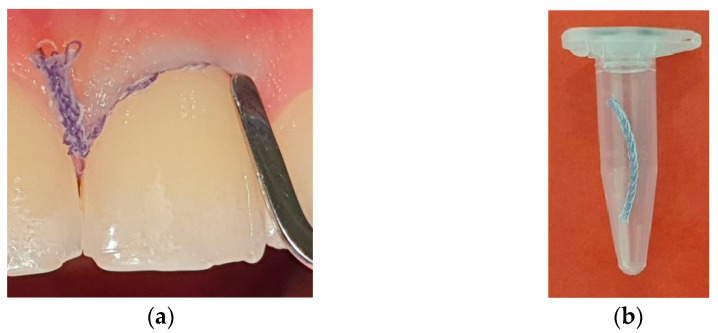
Collection of gingival crevicular fluid samples: (**a**) Retraction cord inserted to gingival crevice and periodontal pocket, respectively; (**b**) Retraction cord sealed in Eppendorf tube.

The GCF sample collection was conducted using a 10 mm long retraction cord (Ultrapak Cleancut, Ultradent, South Jordan, UT, USA). Upon the removal of the supragingival plaque, the teeth and adjacent gingiva were isolated by cotton rolls and dried with air. The retraction cord was carefully inserted into the gingival crevice or the periodontal pocket for 2 min (Figure 1a). The retracting cords were then sealed in 0.5 mL Eppendorf tubes (Figure 1b). Samples contaminated with saliva or blood were discarded.

The GCF samples were analyzed by the newly developed method, described previously in detail [25]. The amount of each GCF sample was obtained by calculating the weight difference of the retraction cord sealed in an Eppendorf tube before and immediately after the collection of GCF. Each cord was extracted with 110 μL of 0.9% saline in an ultrasonic bath (ARGOLAB Digital DU-100 ultrasonic cleaner, Chromservis s.r.o., Prague, Czech Republic). The samples were stored after extraction in 0.5 mL Eppendorf tubes at −80 °C, until analysis was carried out. Then, the centrifugal filter devices were used for the purification and deproteinization of the GCF samples. The supernatant was transferred to a microtitration plate and injected into the ultra-high-performance liquid chromatography (UHPLC) system in connection with mass spectrometry and fluorescence detection.

The stimulated oral fluid was collected using a Salivette® collection kit (Sarstedt AG & Co. KG, Nümbrecht, Germany). The subjects were instructed to chew a cotton swab for sixty seconds. Upon transportation and centrifugation, the samples were stored at −80 °C. The sample preparation technique was carried out under optimized conditions using Microcon centrifugal filter devices (Merck, Darmstadt, Germany) filled with 200 μL thawed oral fluid and 5 μL 3-nitro-L-tyrosine internal standard solution, at a concentration of 50 μmol/L, together with 3 μL 0.5 mmol/L sodium hydroxide. After mixing, the mixture and the supernatant were transferred to a microtitration plate. Subsequent analysis was performed using the high-performance liquid chromatography (HPLC) method with fluorescence detection and diode array detection. The method had been described in detail by Vernerová et al. [26].

The blood samples were drawn from the antecubital vein. The samples were then centrifuged (1600× *g*, 10 min, 4 °C) and the serum was separated and stored at −80 °C [27]. Prior to the analysis, 200 µL of serum was diluted with 100 µL phosphate buffer (15 mmol/L, pH 6.5), deproteinized by 100 µL cooled ethanol (10 min, −25 °C). After centrifugation (14,000× *g*, 10 min), the supernatant was filtered using 0.2 µm micro titration plate filters and vacuum manifold. The filtered solution was injected into the HPLC, coupled with fluorescence detection and diode array detection [27].

Morning urine specimens were collected and stored at −80 °C until analysis. Then, 100 μL of the urine sample was centrifugated (45 s, 12,000× g) and diluted with 1.0 mL of mobile phase (containing disodium ethylenediaminetetraacetic acid 2 g/L). Following this dilution, the samples were filtered using Microtiter, AcroPrep 96 Filter Plate 0.2 μm/350 μL, Pall Life Science (Ann Arbor, MI, USA), and Vacuum manifold Pall Life Science (Ann Arbor, MI, USA) and subsequently injected into a column. Np was measured using the HPLC method with fluoresce and diode array detection, simultaneously, with creatinine [28]. The Np to creatinine ratio (NC) was calculated in order to eliminate different concentrations of urine.

If the concentration of Np in the serum did not reach the limit of quantification, 1.67 nmol/L, the lower limit of quantification [27] was used as the lowest possible analyte value.

### 2.4. Statistical Analysis

The difference in GCF mass, in mg, was then converted into the volume of GCF, in μL, using the density of GCF 1 g/mL [8]. The concentrations of analytes detected by UHLPC were transformed to GCF concentrations because of the different sample dilutions during sample preparation caused by different volumes of GCF samples. The total amount (TA, amount of substance) [8,29] of Np was calculated using the formula:

Total amount [nmol] = concentration [µmol/L] × volume [µL]

For intergroup evaluation, the nonparametric Mann-Whitney U test was used. The Wilcoxon Test was adopted for CGF and OF concentration comparison. The Spearman rank correlation test was adopted for the comparison of GCF concentrations or TA with other matrices. Differences were considered significant at *p* < 0.05. All of the statistical analyzes were performed with MedCalc, version 9.5.2.0 software for Windows OS (MedCalc Software Ltd., Ostend, Belgium). The results are presented as median and interquartile range (IQR).

## 3. Results

### 3.1. Description of Study and Control Group

Characteristics of the study and control groups, including gender, age, full mouth, and sampled tooth evaluation of PD, GR, CAL, PLI, GI and BOP, are displayed in Table 1. All periodontal parameters, with the exception of the full mouth evaluation of GR, were significantly higher in the study group (*p* < 0.05). Nonsurgical periodontal therapy was associated with a significant decrease in all parameters, with the exception of the full mouth and the sampled site GR, where significantly higher values were observed in the before treatment values to the control group values. The DMF index was not significantly different among all groups (*p* > 0.05). The volumes of GCF before periodontal treatment and in the control group were not significantly different [median 5.41 (IQR 3.44–8.42) µL in the study group and 3.86 (IQR 2.37–5.65) µL in the control group, respectively, *p* = 0.095]. Periodontal therapy was associated with a significant decrease in the GCF volume [3.53 (1.85–4.75) µL, *p* = 0.0062]. Following treatment, the GCF volumes were not significantly different from those in control group (*p* = 0.23), see Table 1.

### 3.2. Gingival Crevicular Fluid

**Figure 2:**
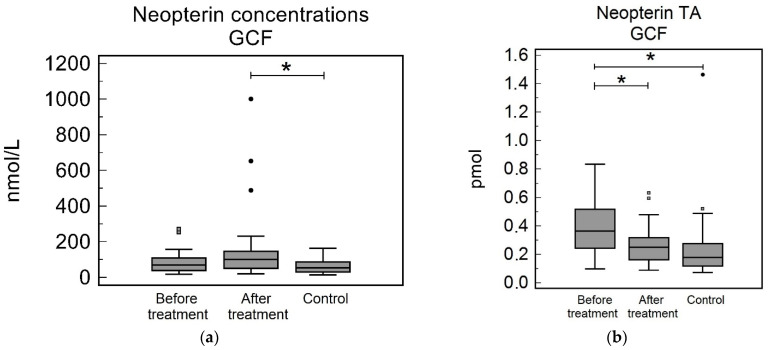
Neopterin in gingival crevicular fluid (GCF): (**a**) Neopterin concentrations (**b**) Neopterin total amount (TA). Neopterin concentrations in study group before treatment were not significantly different to after treatment concentrations (*p* = 0.31) and to concentrations in control group (*p* = 0.32). Neopterin concentrations in study group after periodontal treatment were found to be significantly higher after periodontal treatment to control group (*p* = 0.038). Neopterin TA before treatment were significantly higher to after treatment levels (*p* = 0.024) and to and to levels in control group (*p* = 0.001). Neopterin TA after periodontal treatment were not significantly different to control group (*p* = 0.11). * *p* < 0.05.

The GCF Np concentrations in the study group before treatment and in the control group were not significantly different [69.87 (39.84–108.37) nmol/L and 54.36 (31.02–84.34) nmol/L, respectively, *p* = 0.32]. The Np GCF concentrations in the study group after treatment were not significantly different to the before treatment levels [101.03 (50.84–146.58) nmol/L, *p* = 0.31]; however, they were significantly higher than the control group (*p* = 0.038), see Table 2 and Figure 2a. The TA GCF were significantly higher in the study group before treatment compared to the control group [0.36 (0.24–0.51) pmol and 0.18 (0.12–0.27) pmol, respectively, *p* = 0.001]. The Np TA in the study group after treatment were significantly lower than the before treatment levels [0.25 (0.16–0.32) pmol, *p* = 0.024], but were not significantly different to the control group (*p* = 0.11), see Table 2 and Figure 2b.

### 3.3. Oral Fluid

**Figure 3:**
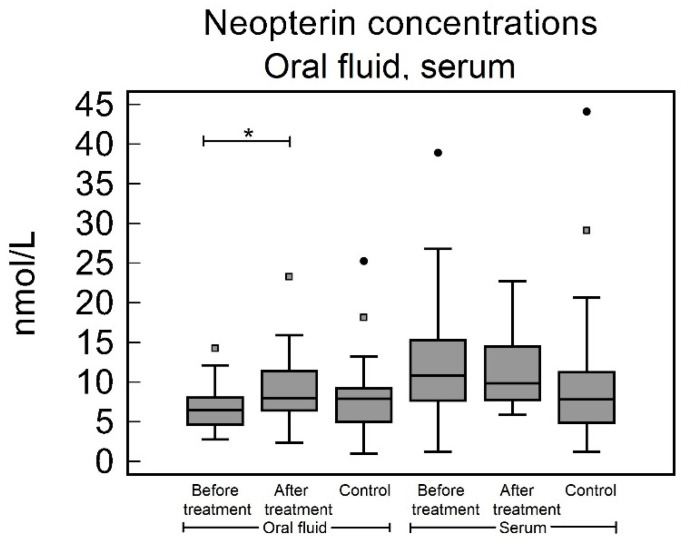
Neopterin concentrations in oral fluid and serum. In oral fluid, Np concentrations were significantly higher after periodontal treatment when compared to pretreatment levels (*p* = 0.02). In oral fluid, when compared with control group, Np concentrations in study group both, before and after treatment, were not significantly different (*p* = 0.27 and 0.5, respectively). In serum no significant difference was observed (*p* = 0. 72, study group before treatment vs. after treatment; *p* = 0.23, study group before treatment vs. control group; *p* = 0.14, study group after periodontal treatment vs. control group. * *p* < 0.05.

No significant difference was found between the oral fluid levels in the study group before treatment and in the control group [6.48 (4.69–8.05) nmol/L and 7.89 (5.00–9.20) nmol/L, respectively, *p* = 0.27]. The oral fluid Np concentrations in the study group after treatment were significantly lower than the before treatment concentrations [8.03 (6.43–11.39) nmol/L, *p* = 0.020], but were not significantly different to the control group (*p* = 0.50), see Table 2 and Figure 3.

### 3.4. Serum

The serum Np levels in the study group before treatment and in the control group were not significantly different [10.83 (7.67–15.34) nmol/L and 7.83 (4.89–11.27) nmol/L, respectively, *p* = 0.23]. The serum Np levels in the study group after treatment were not significantly different to the before treatment concentrations [9.86 (7.69–14.17) nmol/L, *p* = 0.72] and to the control group (*p* = 0.14), see Table 2 and Figure 3.

### 3.5. Urine

**Figure 4:**
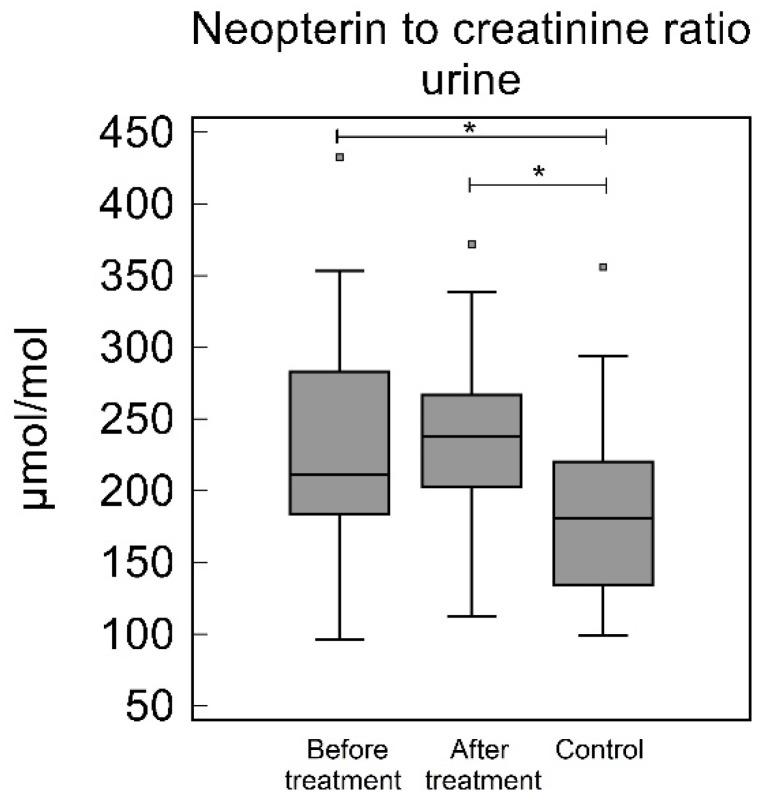
Neopterin to creatinine ratio in urine (NC). NC was significantly higher in study group, both before and after periodontal treatment to control group (*p* = 0.02 and 0.0013, respectively). No significant difference was observed when before treatment NC were compared to after treatment NC (*p* = 0.36) * *p* < 0.05.

The urinary NC in the study group before treatment were significantly higher than the control group [210.96 (183.60–282.91) µmol/mol and 180.59 (133.97–220.22) µmol/mol, respectively, *p* = 0.020]. In the study group after treatment, the NC were not significantly different to the before treatment levels [237.87 (202.46–266.80) µmol/mol, *p* = 0.36], but were significantly higher than the control group (*p* = 0.0013), see Table 2 and Figure 4.

### 3.6. Correlation Analysis

The only significant correlation was found in serum and oral fluid concentrations (*p* = 0.001, ρ = 0.40), see Table 3.

## 4. Discussion

Several previous studies found significantly higher Np concentrations in the GCF [9,11,12] and oral fluid of subjects with periodontitis [15,30]. Periodontal therapy was associated with a decrease in Np levels [9,11,14,18,30]. In our study, when the Np levels in the study group before periodontal treatment were compared with the control group, only GCF TA and urinary NC were significantly higher in the study group. This confirms the impact of macrophages activation on periodontitis. The increase in the Np OF concentrations after treatment and the significant difference in the Np GCF concentrations and the urinary NC between individuals with periodontitis after periodontal therapy and the healthy controls, as well as the significantly higher after treatment Np GCF concentrations, support the role of macrophages in the resolution of periodontal inflammation and the maintenance of periodontal health. This is in accordance with the study performed by Bodur et al. [13].

The presence of the infection in the periodontal tissues leads to the migration of monocytes from the peripheral blood to the periodontal tissue, where they differentiate into monocyte-derived macrophage populations and participate in pathogen clearance and tissue repair [31]. IFN-γ, together with other proinflammatory cytokines, such as tumor necrosis factor alfa (TNF-α), interleukin 1 beta (IL-1β) and IL-6, stimulate macrophages to polarize into M1, which is the proinflammatory phenotype. The M2 phenotype of macrophages, stimulated via IL-4 and IL-13, promotes tissue repair and wound healing [7,32]. They were found in an increased number in the gingival tissue of subjects with gingivitis, where they can play an important role in protecting tissue destruction [7,33]. Changes in the M1/M2 ratio are believed to be associated with the progression of the periodontal lesion [33].

Activated macrophages not only phagocyte periodontal pathogens, but also secrete hydrolytic enzymes, such as collagenase, different matrix metalloproteinases (MMP, e.g., 8, 9, 12) that cleave different compounds from connective tissue [8,33,34]. M1 macrophages also release a large amount of superoxide anions and oxygen and nitrogen radicals [7,35]. M1 macrophages can also help the progression of inflammatory disease through the production of proinflammatory cytokines that upregulate cell regulation and induce proinflammatory cytokines such as TNF-α, IL-1, IL-6, IL-8 and IL-12 [33,34].

In human macrophages, GTP is metabolized to 7,8-dihydronepterintriphosphate via cyclohydrolase I. This enzyme is primarily upregulated through IFN-γ, while IFN-α and bacterial lipopolysaccharides participate in upregulation to a lesser degree [36,37]. Consequently, 7,8-dihydronepterintriphosphate is then converted to 7,8-dihydroneopterin by nonspecific phosphatases [36]. Due to the low expression of 6-pyruvoyltetrahydropterin synthase in human macrophages, 7,8-dihydroneopterin is the main product of GTP metabolism in human macrophages; 7,8-dihydroneopterin is transported through the cytoplasmic membrane and is oxidized nonenzymatically to Np. As a potent antioxidant for radical elimination, 7,8-dihydroneopterin protects monocytes and macrophages from oxide hydroxyl and peroxyl radicals, hypochlorous acid, and possibly superoxide [36,38,39].

GCF is a transudate or exudate of human plasma and depends on vascular permeability, which increases with the inflammation of the periodontal tissue [40,41]. Secreted in site of inflammation gives the best information about the ongoing inflammatory condition [40,42,43]. In our study, the evaluation of Np concentrations and TA revealed different results. The Np concentrations were not significantly different or were significantly higher when the study group after periodontal treatment was compared to the control group. The TA of Np was found to be significantly higher in the study group before periodontal treatment than the control group or the study group after periodontal treatment. This is in accordance with the results and the recommendation of Ozmeric et al. [8], who proposed TA Np as more valuable for assessing Np levels because the increased GCF flow in inflamed periodontal tissues may negatively influence Np concentration. On the other hand, the use of TA is controversial. The resting volume of GCF in the periodontal pocket increases with PPD. When recurrent GCF collection is not adopted, TA reflects more resting volume than GCF flow [29]. Moreover, the GCF concentrations are more consistent with our results obtained in oral fluid or urine samples.

Oral fluid is an attractive body fluid due to its simple collection. Several analytes were found to correlate with their serum levels [26]. In periodontitis, several inflammatory biomarkers were found to increase in OF and to evaluate the ‘whole mouth’ inflammatory status, while GCF is site specific [44]. OF samples revealed a significant increase in Np levels after periodontal therapy. This is in contradiction with previous studies [8,13,14,30]. Ozmeric et al. [8] suggested that Np in OF may originate mainly from the salivary gland as a result of nitric oxide production. The results of our correlation analysis suggest that the main source of Np in oral fluid is serum.

The serum Np concentrations did not reveal any significant differences. This is in contradiction with previous studies [11,14,18]. This may be explained by the low impact of periodontitis on systemic Np levels or, by different analytic methods, such as the enzyme linked immunosorbent assay, that were adopted for sample analysis.

Np is excreted via the kidney in an unchanged form [37]. The significantly higher NC ratio in the study group before treatment than in the control group confirms the impact of periodontitis on Np levels. The significantly higher NC in the study group after nonsurgical periodontal treatment compared to the control group are in accordance with Bodur et al. [13], who found significant increases in NC after treatment in the study group; however, these results may be negatively influenced the by low number (8) of individuals in the study group.

Our study had some limitations. The overdilution of the GCF samples during sample preparation may influence the accuracy of the results. Although the sampled tooth was dried and isolated by cotton swabs, the contamination of the GCF samples by oral fluid could not be entirely avoided. Moreover, this study was performed in a European (Czech) population and cannot be generalized. Despite the age match across the study and control groups, given the high incidence of atherosclerosis in older individuals, diseases not recognized in this study may affect the Np levels recorded. Finally, the simultaneous evaluation of multiple inflammatory biomarkers, such as Il-1 or TNF-α, could provide more accurate information about local and systemic disease activity.

## 5. Conclusions

Np levels are involved in periodontitis. Our results confirm the impact of cellular immunity on periodontitis and the resolution of periodontal inflammation Np in oral fluid, which probably originate from serum.

## Figures and Tables

**Table 1:** Periodontal status of subjects included in the study.

	Study Group before Treatment Median (IQR)	Study Group after Treatment Median (IQR)	Control Group Median (IQR)	Before vs. Control *p*-Value	Before vs. after *p*-Value	After vs. Control *p*-Value
Age	50 (47–54)	50 (47–54)	46 (44–54)	0.263		
Full mouth evaluation						
PPD	3.91 (3.55–4.93)	1.70 (1.30–1.94)	1.17 (1.125–1.23)	<0.001	<0.001	<0.001
GR	0.17 (0–0.37	0.213 (0.045–1.937)	0.036 (0.0062–0.77)	0.053	0.015	<0.001
CAL	4.35 (3.90–5.01)	1.86 (1.56–2.51)	1.20 (0–0.083)	<0.001	<0.001	<0.001
PLI	2.39 (1.02–2.95)	0.095 (0.052–0.134)	0.018 (0–0.083)	<0.001	0.006	<0.001
GI	2.60 (1.82–2.95)	0.082 (0.048–0.153)	0.090 (0.060–0.12)	<0.001	<0.001	1.00
BOP %	100 (95.65–100)	0.00 (0.00–0.15)	0.00 (0.00–0.00)	<0.001	<0.001	0.23
Sampled teeth evaluation						
PPD	5.67 (5–6.5)	2.33 (1.62–2.71)	1 (1–1.67)	<0.001	<0.001	<0.001
GR	0.17 (0–0.37)	0.33 (0.00–0.92)	0.00 (0.00–0.00)	0.004	<0.001	0.001
CAL	6 (5.24–7)	2.67 (1.63–3.21)	1.17 (1–2.5)	<0.001	<0.001	<0.001
PLI	2.5 (1.33–3)	0.17 (0.00–0.17)	0.00 (0.00–0.00)	<0.001	<0.001	0.001
GI	3 (2.17–3)	0.083 (0.00–0.167)	0.00 (0.00–0.33)	<0.001	<0.001	0.035
BOP %	1 (0.83–1)	0.00 (0.00–0.00)	0.00 (0.000.00)	<0.001	<0.001	>0.999
DMF index *	15 (12–19)	15 (12–20)	13 (8–16)	0.17	0.65	0.13

* Without 3rd molars; IQR—interquartile range, PPD—periodontal pocket depth, GR—gingival recession, CAL—clinical attachment loss, GI—gingival index, BOP—bleeding on probing, DMF index—decayed, missing, filled index.

**Table 2:** Neopterin levels in gingival crevicular fluid, oral fluid, serum and urine.

	Study Group before Treatment	Study Group after Treatment	Control Group	Before vs. Control	Before vs. After	After vs. Control
Sample	Median (IQR)	Median (IQR)	Median (IQR)	*p*-Value	*p*-Value	*p*-Value
GCF Conc. [nmol/L]	69.87 (39.84–108.37)	101.03 (50.84–146.58)	54.36 (31.02–84.34)	0.322	0.31	0.038
GCF TA [pmol]	0.36 (0.24–0.51)	0.25 (0.16–0.32)	0.18 (0.12–0.27)	0.001	0.024	0.11
Oral fluid [nmol/L]	6.48 (4.69–8.05)	8.03 (6.43–11.39)	7.89 (5.00–9.20)	0.27	0.020	0.50
Serum [nmol/L]	10.83 (7.67–15.34)	9.86 (7.69–14.17)	7.83 (4.89–11.27)	0.23	0.72	0.14
Urine NC [µmol/mol]	210.96 (183.60–282.91)	237.87 (202.46–266.80)	180.59 (133.97–220.22)	0.020	0.36	0.0013
GCF vol. [µL]	5.41 (3.44–8.42)	3.53 (1.85–4.75)	3.86 (2.37–5.65)	0.095	0.0062	0.23

IQR—interquartile range, GCF—gingival crevicular fluid, Conc.—concentrations, TA—total amount, NC—Np to creatinine ratio, vol.—volume.

**Table 3:** Results of correlation analysis among different biological fluid.

	*p*-Value	ρ
GCF concentration. vs. oral fluid	0.35	0.11
GCF concentration. vs. serum	0.51	0.078
GCF concentration. vs. urinary NC	0.11	0.19
GCF total amount vs. oral fluid	0.18	0.16
GCF total amount vs. serum	0.83	0.025
GCF total amount vs. urinary NC	0.13	0.157
Serum vs. oral fluid	0.001	0.40

ρ—Spearman’s rank coefficient, GCF—gingival crevicular fluid, NC—Np to creatinine ratio.

## Data Availability

The data presented in this study are available on request from the corresponding author. The data are not publicly available due to personal data protection.
